# After-Effects of Intermittent Theta-Burst Stimulation Are Differentially and Phase-Dependently Suppressed by α- and β-Frequency Transcranial Alternating Current Stimulation

**DOI:** 10.3389/fnhum.2021.750329

**Published:** 2021-11-12

**Authors:** Katsuya Ogata, Hisato Nakazono, Takuro Ikeda, Shin-ichiro Oka, Yoshinobu Goto, Shozo Tobimatsu

**Affiliations:** ^1^Department of Pharmacy, School of Pharmaceutical Sciences at Fukuoka, International University of Health and Welfare, Okawa, Japan; ^2^Department of Occupational Therapy, Faculty of Medical Science, Fukuoka International University of Health and Welfare, Fukuoka, Japan; ^3^Department of Physical Therapy, School of Health Sciences, Fukuoka International University of Health and Welfare, Fukuoka, Japan; ^4^School of Medicine, International University of Health and Welfare, Naritaa, Japan; ^5^Department of Orthoptics, Faculty of Medical Science, Fukuoka International University of Health and Welfare, Fukuoka, Japan

**Keywords:** transcranial alternating current stimulation, transcranial magnetic stimulation, primary motor cortex, motor evoked potentials, intermittent theta burst stimulation, phase dependency, combined stimulation

## Abstract

Intermittent theta-burst stimulation (iTBS) using transcranial magnetic stimulation (TMS) is known to produce excitatory after-effects over the primary motor cortex (M1). Recently, transcranial alternating current stimulation (tACS) at 10 Hz (α) and 20 Hz (β) have been shown to modulate M1 excitability in a phase-dependent manner. Therefore, we hypothesized that tACS would modulate the after-effects of iTBS depending on the stimulation frequency and phase. To test our hypothesis, we examined the effects of α- and β-tACS on iTBS using motor evoked potentials (MEPs). Eighteen and thirteen healthy participants were recruited for α and β tACS conditions, respectively. tACS electrodes were attached over the left M1 and Pz. iTBS over left M1 was performed concurrently with tACS. The first pulse of the triple-pulse burst of iTBS was controlled to match the peak (90°) or trough (270°) phase of the tACS. A sham tACS condition was used as a control in which iTBS was administered without tACS. Thus, each participant was tested in three conditions: the peak and trough of the tACS phases and sham tACS. As a result, MEPs were enhanced after iTBS without tACS (sham condition), as observed in previous studies. α-tACS suppressed iTBS effects at the peak phase but not at the trough phase, while β-tACS suppressed the effects at both phases. Thus, although both types of tACS inhibited the facilitatory effects of iTBS, only α-tACS did so in a phase-dependent manner. Phase-dependent inhibition by α-tACS is analogous to our previous finding in which α-tACS inhibited MEPs online at the peak condition. Conversely, β-tACS reduced the effects of iTBS irrespective of its phase. The coupling of brain oscillations and tACS rhythms is considered important in the generation of spike-timing-dependent plasticity. Additionally, the coupling of θ and γ oscillations is assumed to be important for iTBS induction through long-term potentiation (LTP). Therefore, excessive coupling between β oscillations induced by tACS and γ or θ oscillations induced by iTBS might disturb the coupling of θ and γ oscillations during iTBS. To conclude, the action of iTBS is differentially modulated by neuronal oscillations depending on whether α- or β-tACS is applied.

## Introduction

Transcranial alternating current stimulation (tACS) is a non-invasive brain stimulation (NIBS) method that uses alternating current over the scalp, typically without a direct current shift. An early report revealed that motor learning was modulated after 2–10 min of 0.4 mA tACS at 10 Hz but not 1, 15, 30, or 45 Hz, while motor evoked potentials (MEPs) were not modulated after stimulation at any of these frequencies (Antal et al., 2008). Following this study, the effects of tACS over the primary motor cortex (M1) have been investigated and their dependency on tACS frequency has been reported (Feurra et al., 2011). Specifically, tACS at 20 Hz but not 5, 10, or 40 Hz with 1 mA was effective during stimulation. These studies indicate that tACS effects depend on the stimulation intensity as well as duration and frequency. Although the underlying mechanisms that produce this frequency dependency have not been established, the entrainment of cortical oscillations may be involved (Herrmann et al., 2013). Subsequently, the effects have been suggested to depend on the phase (Guerra et al., 2016; Nakazono et al., 2016; Raco et al., 2016), which is in line with the entrainment of oscillations.

Theta-burst stimulation (TBS) is a patterned form of transcranial magnetic stimulation (TMS) in which triple-pulse bursts (typically 50 Hz) are repeated at 5 Hz (Huang et al., 2005). Continuous and intermittent TBS [cTBS, Intermittent theta-burst stimulation (iTBS)] result in cortical inhibition and excitation, respectively. The mechanisms underlying these TBS after-effects are not fully understood; however, long-term potentiation (LTP) or long-term depression (LTD) through N-methyl-D-aspartate receptor or γ-aminobutyric acid have been suggested to be important factors (Cárdenas-Morales et al., 2010; Rounis and Huang, 2020). The ability to modulate cortical excitability with a short stimulation duration popularized TBS, and it has been applied widely not only to the motor cortices, but also to other brain regions as a means to combat symptoms of psychiatric conditions such as depression (Blumberger et al., 2018; Rounis and Huang, 2020). However, the effects of TBS have been shown to be variable (Hamada et al., 2013), as with other methods of NIBS (Wiethoff et al., 2014; Guerra et al., 2020a). Latency differences resulting from different TMS current directions, which involve I-wave recruitment, have been proposed to contribute to the variability (Hamada et al., 2013), while cortical oscillations recorded by electroencephalography (EEG) have been shown to reflect cortical excitability indexed by MEP amplitudes (Khademi et al., 2018; Zrenner et al., 2018; Ogata et al., 2019). Thus, variability in cortical oscillations might influence NIBS effects. Indeed, repeated burst pulses of TMS that were synchronized to the peak phase of α-band EEG at the central region, which reflects endogenous cortical oscillations around the sensorimotor region, resulted in post-intervention MEP facilitation (Zrenner et al., 2018). Accordingly, we hypothesized that coupling iTBS with oscillations entrained by a specific phase of tACS could lead to more apparent after-effects. In a previous study, iTBS combined with γ-band (70 Hz) tACS resulted in an enhanced iTBS effect, while β-band (20 Hz) tACS did not modulate the iTBS effect in either direction (Guerra et al., 2018). However, the tACS phase was not aligned with the TMS pulses in that study. In our recent study (Nakazono et al., 2021), synchronized tACS with repetitive paired-pulse stimulation (rPPS) enhanced M1 excitability more than rPPS alone at the peak phase of 20-Hz tACS, but not at the trough phase. Conversely, 10-Hz tACS did not facilitate rPPS after-effects at either the peak or trough phases. Thus, the effects of tACS with rPPS are phase and frequency dependent. In this study, we investigated the synchronized effects of α- and β-frequency tACS when combined with iTBS.

## Materials and Methods

### Participants

Eighteen healthy volunteers (men = 10, women = 8; age: 20–24 years) were recruited for the 10-Hz (α) tACS experiment and thirteen (men = 8, women = 5; age: 20–35 years) for the 20-Hz (β) tACS experiment. One individual participated in both experiments. The sample size was determined based on a recent systematic review that considered 82 iTBS studies with samples ranging from 2 to 77 participants (mean ± SD: 13.2 ± 10.8) (Chung et al., 2016). Thus, the current sample sizes were within the average range. All participants were right-handed by self-report, and none had a history of neuropsychological disorders. All participants gave their written informed consent following an explanation of the experiments in accordance with the Declaration of Helsinki. This study was approved by the Ethics Committee of Kyushu University and the International University of Health and Welfare.

### Motor Evoked Potentials

Motor evoked potentials were recorded as in our previous studies (Nakazono et al., 2016; Hayashi et al., 2019; Ogata et al., 2019). In brief, participants sat in a comfortable chair and kept their right hands relaxed. They were asked to keep their eyes open. MEPs were recorded from their right first dorsal interosseus muscle. Recordings were band-pass filtered between 10 and 3,000 Hz and sampled at 10 kHz. MEP data from 250 ms before TMS onset until 250 ms after TMS onset were stored on a Windows PC using Multiscope PSTH software ver. 1.7 (MedicalTry System, Tokyo, Japan) for offline analysis. Electromyographies (EMGs) were shown to the participants on a monitor as visual feedback. Single-pulse TMS was delivered with a monophasic Magstim 200 (Magstim Co., Ltd., Whitland, United Kingdom) with a figure-of-eight coil that was 70 mm in diameter. The TMS coil was placed over the left M1 hot spot with the handle pointing posterolaterally to approximately 45° from the midline. To ensure that the positions of the coils were constant throughout each session, their position and orientation were marked in pen on plastic wrap covering the scalp. The TMS intensity was adjusted to obtain 0.5–1.5 mV MEPs, which occurred at 53.1 ± 9.7% of maximum stimulator output (mean ± SD) for the α-tACS experiment and 53.6 ± 7.7% for the β-one. Twenty-four MEPs were obtained for each recording with an interstimulus interval of 5–7 s.

### Intermittent Theta-Burst Stimulation

Intermittent theta-burst stimulation pulses were delivered using a Magstim SuperRapid system (Magstim Co., Ltd., Whitland, United Kingdom) with a figure-of-eight coil that was 70 mm in diameter. Three 50-Hz pulses with a 20-ms interval were repeated every 200 ms (5 Hz) for 2 s with an 8-s pause. Six hundred pulses (200 s) were delivered over the left M1 “hot spot” in each session. The stimulus intensity was set to 80% of the active motor threshold (Huang et al., 2005), which was determined as the minimum intensity needed to obtain 200 μV MEPs with weak contraction of the target muscle in at least 5 of 10 trials (Groppa et al., 2012). The active motor threshold was 50.1 ± 6.7% (mean ± SD) for the α-tACS experiment and 50.6 ± 6.6% for the β-one, respectively.

### Transcranial Alternating Current Stimulation

**FIGURE 1:**
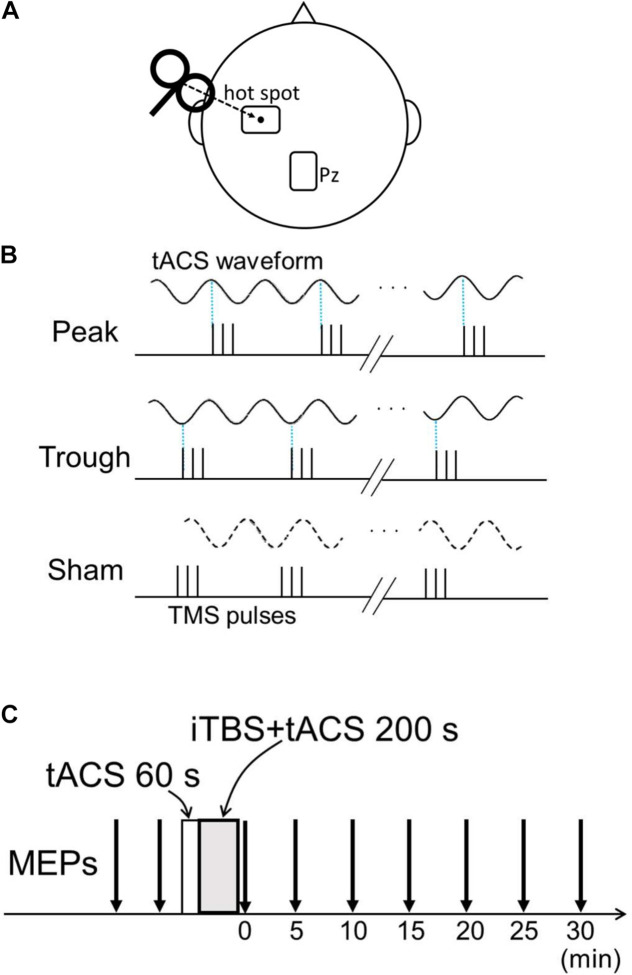
Experimental setup. **(A)** Transcranial alternating current stimulation (tACS) electrode position and transcranial magnetic stimulation (TMS) coil configuration. The target electrode was placed over the left M1 “hot spot,” and TMS was delivered over the target electrode. The reference electrode was placed over Pz. **(B)** Three conditions for the combined tACS/intermittent theta-burst stimulation (iTBS) pulses. On the basis of the tACS phase, the first of the three pulses was adjusted to 90° for the peak condition and to 270° for the trough condition. For the sham condition, tACS only lasted 20 s and was terminated before iTBS began. Thus, no tACS current flowed during iTBS. **(C)** Time course of the experiment. Baseline motor evoked potentials (MEPs) were obtained from two sessions comprising the recording of 24 MEPs before the combined stimulation. tACS began before iTBS and continued after iTBS onset for 200 s. After the intervention, 24 MEPs were recorded every 5 min for 30 min.

Transcranial alternating current stimulation was administered as in our previous studies (Nakazono et al., 2016, 2021). Two 5 × 7 cm self-adhesive electrodes (PALS electrodes, Axelgaard Manufacturing Co., Ltd., Fallbrook, CA, United States) were attached with electrically conductive gel (Gelaid, Nihonkohden, Tokyo, Japan) over the left M1 hot spot and Pz according to the international 10–20 system (Figure 1A). tACS was delivered using a battery-driven current stimulator (DC Stimulator-Plus, NeuroConn GmbH, Ilmenau, Germany) with an intensity of 1 mA. The electrode size, position, and current intensity were chosen based on previous studies (Feurra et al., 2011; Helfrich et al., 2014; Guerra et al., 2016; Nakazono et al., 2016, 2021). The tACS duration was 260 s, and the stimulation began 60 s before iTBS onset and lasted until iTBS offset. The tACS current was ramped up and down in 5-s increments to reduce skin sensations.

### Procedures

Intermittent theta-burst stimulation was synchronized with α- and β-tACS in separate experiments. All participants completed three conditions in each experiment: peak (90°), trough (270°), and sham, where the sham condition comprised iTBS without tACS and was used as a control. For the first two conditions, the timing of the first iTBS pulse was controlled to match either the peak or trough phase of the tACS (Figure 1B). Because there was a slight delay of about 10 ms between the tACS phase and iTBS pulse in the combined 260 s stimulation without adjustment, we calculated the precise iTBS pulse time accordingly, enabling us to match the iTBS pulse with the tACS phase. The tACS waveforms and TMS pulses were recorded during combined stimulation, and synchronization between the iTBS pulse and tACS phase was confirmed. In the sham condition, tACS was only administered for 20 s, and thus current did not flow during iTBS. Baseline MEP amplitudes were obtained in two 24-MEP sessions before the combined stimulation. After the combined stimulation, 24 MEPs were obtained for 30 min at 5-min intervals (i.e., 0, 5, 10, 15, 20, 25, 30 min after stimulation; Figure 1C). The different conditions took place at least 2 days apart, and the order of the three conditions (peak, trough, and sham) was randomized and counterbalanced across the participants. The order of the conditions was blinded only to the subjects; thus, the experiment had a single-blind, cross-over design.

### Data Analysis

Motor evoked potential waveforms were visually checked, and trials with artifacts (about 50 μV or larger) during the 100 ms before TMS pulse onset were discarded. Fewer than 17% (4/24) trials were discarded in each session. Peak-to-peak amplitudes were measured and log-transformed to normalize their distribution, and then averaged for each recording (Nielsen, 1996; Avenanti et al., 2006; Feurra et al., 2011; Guerra et al., 2016; Nakazono et al., 2016; Borgomaneri et al., 2020). Mean MEP amplitudes after combined tACS/iTBS were normalized by subtracting the averaged amplitudes over the two baseline MEP sessions, as in previous studies (Avenanti et al., 2006; Goldsworthy et al., 2016; Borgomaneri et al., 2020). This was done because we intended to estimate the after-effects of tACS over those of iTBS, i.e., to compare MEPs modulated by combined iTBS and tACS (peak or trough) with those modulated by iTBS only (sham). The three stimulation conditions were compared using a linear mixed-effect (LME) model with fixed effects of time (0–30 min after combined stimulation) and phase condition (peak, trough, and sham), as well as the random effects of participant. When a main effect of phase condition was significant, pairwise comparisons were performed with the Holm–Bonferroni correction. A *p*-value less than 0.05 was considered to be significant. Error bars represent the standard error of the mean (SEM) throughout the study. The data from the α- and β-tACS experiments were analyzed separately. The baseline MEP amplitudes were also compared using a LME model with fixed effects of phase condition and the random effects of participant. Statistical analyses were carried out using R (R Core Team, 2020). Untransformed MEP amplitudes were also analyzed for comparison of the transformed MEP data.

## Results

**FIGURE 2:**
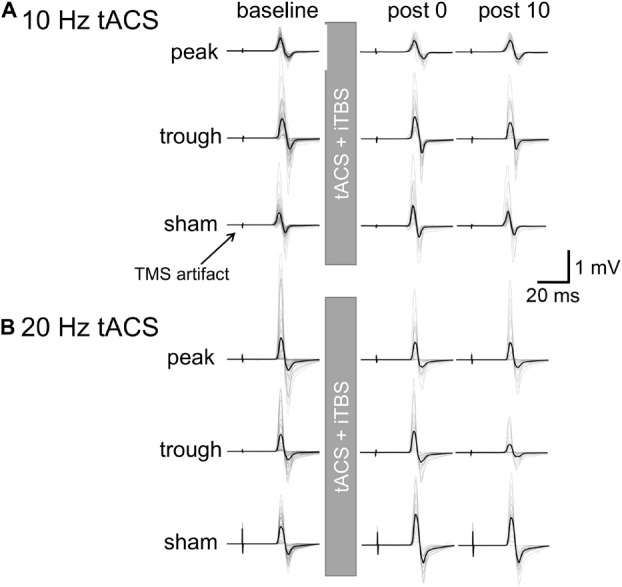
Motor evoked potential waveforms from a representative participant for iTBS with α- or β-tACS. MEP waveforms are shown for the baseline and 0 and 10 min after the combined stimulation. Thick lines indicate the mean waveform calculated from 24 trials in each session. Thin lines indicate each of the 24 trials. **(A)** iTBS with α-tACS. MEPs increased after the trough and sham conditions but were suppressed after the peak condition. **(B)** iTBS with β-tACS. Compared with the sham condition, MEPs were inhibited after both the peak and trough conditions, which differed from the iTBS/α-tACS results. For actual data analysis, MEP amplitudes were measured for each trial, not for average waveforms.

**FIGURE 3:**
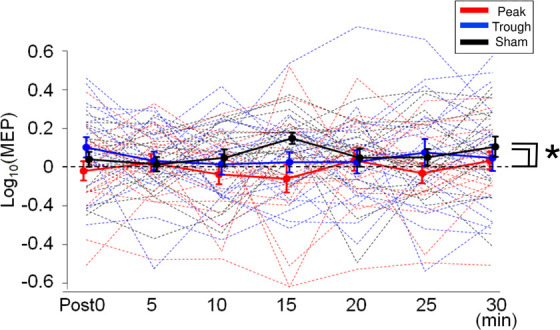
Mean MEP amplitudes after iTBS/α-tACS. MEP amplitudes were log-transformed and normalized by the baseline amplitudes. Accordingly, positive values indicate enhancement after the stimulation while negative values imply MEP suppression. Thick solid lines represent mean amplitudes while dotted lines indicate the MEP amplitudes for each subject. MEP amplitudes after the sham and trough conditions were enhanced continuously after stimulation, while those after the peak condition were often suppressed. Accordingly, MEP amplitudes for the peak condition were lower than those for the sham and trough conditions. Error bars indicate standard errors of the mean (SEM). *p < 0.05.

Motor evoked potential waveforms from a representative participant are shown in Figure 2A for the α-tACS experiment. MEPs were enhanced after the sham (iTBS only) and trough conditions but not after the peak condition. The baseline amplitudes for α- and β-tACS were not significantly different (*p* = 0.52 and 0.32, respectively). Thus, MEP amplitudes that had been subtracted from baseline were used in the following analyses (as described in the Methods). The changes of mean log-transformed MEP amplitudes for α-tACS/iTBS from baseline values are shown in Figure 3. The synchronized stimulation resulted in phase-dependent effects. Mean MEP amplitudes for the sham and trough conditions were larger after α-tACS/iTBS (positive values). However, those for the peak condition were often lower than baseline (negative values). Therefore, MEP amplitudes were lower for the peak condition than for the trough or sham conditions. A LME model indicated a main effect of tACS condition (*F*_(__2_,_357__)_ = 4.74, *p* = 0.009, η_*p*_^2^ = 0.03). Furthermore, we found significant differences between the peak and sham conditions (*F*_(__1_,_232__)_ = 10.4, *p* = 0.004, 95% confidence interval (CI) = 0.029 – 0.118) and between the peak and trough conditions (*F*_(__1_,_232__)_ = 5.15, *p* = 0.048, 95% CI = 0.006 – 0.103), but not between the trough and sham conditions (*F*_(__1_,_232__)_ = 0.57, *p* = 0.45). These results indicate that synchronized tACS in the peak condition inhibited the effects of iTBS relative to the sham and trough conditions. The intercept of the data produced by the LME model, in which the sham stimulation was the control, was 0.05 ± 0.03 (*p* = 0.1). Thus, the MEP amplitudes after the sham condition were above zero, although this enhancement was not confirmed statistically. Of note, these results were obtained by the log-transformed MEP amplitudes followed by subtracting baseline values as described in the section “Data Analysis,” but not by the raw MEP amplitudes.

**FIGURE 4:**
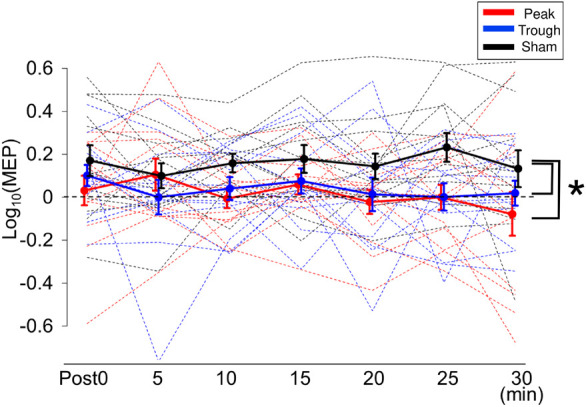
Mean MEP amplitudes after iTBS/β-tACS. MEP amplitudes were processed in the same manner as that described in Figure 3. MEP amplitudes for the sham condition were clearly enhanced after the intervention, whereas those for the peak and trough conditions were not significantly modulated. Consequently, MEP amplitudes for the peak and trough conditions were lower than those for the sham condition. Error bars indicate SEM. *p < 0.05.

Figure 2B shows the representative MEP waveforms for the β-tACS experiment. MEPs were enhanced after the sham condition but not after the peak or trough conditions. As seen in Figure 4, β-tACS with iTBS modulated the mean MEP amplitudes, but not in the same way as with α-tACS. β-tACS/iTBS erased the iTBS effect, and MEP amplitudes in the peak and trough conditions remained unenhanced. A LME model revealed a main effect of the tACS condition (*F*_(__2_,_257__)_ = 14.0, *p* < 0.001, η_*p*_^2^ = 0.10). Further analysis showed a significant difference between the sham and peak conditions (*F*_(__1_,_167__)_ = 28.0, *p* < 0.001, 95% CI = 0.092 – 0.201) and between the sham and trough conditions (*F*_(__1_,_167__)_ = 17.3, *p* < 0.001, 95% CI = 0.065 – 0.181), but not between the peak and trough conditions (*F*_(__1_,_167__)_ = 0.62, *p* = 0.43). The intercept of the LME model, in which the sham was the control, was 0.18 ± 0.04 (*p* < 0.001), which indicates that the MEPs were enhanced in the sham condition. In sum, iTBS-induced enhancement of M1 excitability was inhibited when the stimulation was synchronized with 20-Hz tACS. This was irrespective of the tACS phase, which was unlike the phase-dependent inhibition seen with α-tACS synchronization. Again, these results were not found by the raw MEP amplitude analysis as in α-tACS experiment.

## Discussion

We investigated the effect of synchronizing α- and β-tACS with iTBS on M1 excitability. Our results revealed that (1) α-tACS suppressed iTBS in the peak condition, but not in the trough condition, and that (2) β-tACS inhibited iTBS-induced enhancement regardless of the tACS phase. Thus, we have demonstrated for the first time that α- and β-tACS modulate iTBS after-effects in both frequency and phase-dependent manners.

### Online Effects of Transcranial Alternating Current Stimulation

Online effects of tACS have been reported to modulate cortical excitability in a frequency-dependent manner. For instance, Feurra et al. (2011) reported that while β-tACS effectively enhanced M1 excitability, other frequencies (5, 10, or 40 Hz) did not. Our previous study (Nakazono et al., 2016) also revealed frequency and phase-dependent effects of M1-tACS on the α and β bands: tACS at the peak phase was effective in differentiating the facilitatory effect of 20-Hz tACS from the inhibitory effect of α-tACS. Previous studies have reported online phase-dependent MEP modulation by β-tACS (Guerra et al., 2016; Raco et al., 2016; Schilberg et al., 2018), which is in line with the entrainment of cortical oscillations (Thut et al., 2011; Herrmann et al., 2013). However, the relationship between phase and MEP amplitudes was not consistent among previous studies. Taken together, the online effects of tACS are clear for α/β frequencies.

### Combined Transcranial Alternating Current Stimulation and Intermittent Theta-Burst Stimulation

Following the significant online tACS effects discussed above, iTBS combined with γ-tACS was reported to enhance the effects of iTBS over M1, but the same was not true for β-tACS (Guerra et al., 2018). In that study, the tACS phase was not synchronized with the iTBS pulses. Thus, we assumed that β-tACS synchronized with iTBS might reveal facilitatory effects beyond what is seen after M1 iTBS. Indeed, we recently found that the facilitatory after-effects of rPPS over M1 were more pronounced with β-ACS when the TMS pulses were synchronized to the peak phase but not the trough phase (Nakazono et al., 2021). Conversely, α-tACS did not significantly modulate the effects of rPPS. The inhibitory effect of cTBS on M1 was found to be greater with α-tACS when the TBS pulses were aligned to the trough phase than when they were aligned to the peak phase (Goldsworthy et al., 2016). Adding to these previous studies, our current results indicate that synchronization of α-tACS with iTBS suppressed M1 excitability compared with iTBS delivered in a phase-dependent manner. Specifically, MEP amplitudes in the peak condition were lower than those in the trough as well as the sham condition. Because α-tACS synchronized at the peak phase tended to suppress single-pulse MEPs (Nakazono et al., 2016), it is likely that the effect of iTBS was suppressed by the inhibitory effect of peak-phase α-tACS, but not trough-phase α-tACS. It has been proposed that α oscillations reflect pulsed inhibition or top-down inhibitory control, depending on their amplitudes and phases (Klimesch et al., 2007; Jensen and Mazaheri, 2010; Mazaheri and Jensen, 2010). In a previous study, depressive cTBS effects were enhanced by trough-phase α-tACS (Goldsworthy et al., 2016), which seems to contradict the current results. If peak-phase α-tACS has inhibitory effects *via* pulsed inhibition, trough-phase α-tACS could lead to less inhibitory or facilitatory effects. Cortical modulation by iTBS and cTBS may be derived from LTP and LTD-like mechanisms. While high-frequency bursts may be necessary to induce LTP-like modulation, overstimulation could reduce or reverse LTP-like effects (Wischnewski and Schutter, 2015). Thus, cTBS could cause LTD-like effects through overstimulating bursts, whereas 2-s iTBS pulses with 8-s intervals could induce cortical potentiation. In these circumstances, more efficient bursts of cTBS synchronized with the trough phase of tACS would lead to more overstimulation by continuous bursts, resulting in greater depression of cortical excitability. In contrast, less efficient iTBS bursts synchronized with the peak phase of tACS would disturb the induction of iTBS effects, as was observed in the current study.

In contrast to α-tACS, β-tACS suppressed the effects of iTBS regardless of the tACS phase. Although the reasons for this are unclear, given that unsynchronized β-tACS did not modulate the iTBS after-effects (Guerra et al., 2018), the synchronization of β-tACS appears to be critical for inhibiting iTBS over M1. β oscillations are thought to originate in the motor cortices, whereas α oscillations are thought to be generated by the somatosensory cortices (Salmelin et al., 1995). β oscillations over M1 have been suggested to reflect idling rhythm, response inhibition, or maintenance of the *status quo* (Engel and Fries, 2010). In pathological conditions such as Parkinson disease, exaggerated coupling of the local field potentials has been reported between β phase and γ oscillations in M1 (de Hemptinne et al., 2013). Therefore, excessive coupling of β-tACS and γ oscillations induced by iTBS might result in the suppression of iTBS effects. One of the proposed mechanisms through which tACS is thought to affect cortical excitation is spike-timing-dependent plasticity, in which the interaction between ongoing oscillatory activity and tACS is important for tACS effectiveness (Zaehle et al., 2010). The coupling of θ and γ rhythms is considered important for cortical functions such as cognitive processing (Canolty et al., 2006; Lisman, 2010) and is also assumed to be linked to the induction of TBS effects (Rounis and Huang, 2020). Taken together, unlike γ-tACS, entraining β oscillations with tACS might interfere with the coupling of θ and γ rhythms, which hampers LTP induction during iTBS (Guerra et al., 2018).

### After-Effects of Transcranial Alternating Current Stimulation

Although our current results could be derived from an interaction between tACS and iTBS, another explanation involves additive tACS after-effects. tACS after-effects have been consistently reported in studies using much higher stimulation frequencies (140 Hz and 250 Hz tACS; Moliadze et al., 2010, 2012; Inukai et al., 2016; Guerra et al., 2020b). α- and β-tACS also induce after-effects on visual (Zaehle et al., 2010; Neuling et al., 2013; Vossen et al., 2015; Kasten et al., 2016; Nakazono et al., 2020) and auditory (Ahn et al., 2019; Wang et al., 2020) neurophysiological functions. Conversely, α- and β-tACS have not consistently been reported to produce after-effects on M1. Rjosk et al. (2016) reported that 20-Hz tACS over M1 for 20 min did not modulate MEP amplitudes or induce intracortical or interhemispheric inhibition. Several other studies have also failed to observe effects after α- and/or β-tACS (Antal et al., 2008; Feurra et al., 2011, 2019; Wach et al., 2013; Nakazono et al., 2016; Pozdniakov et al., 2021), yet other studies have observed effects after β-tACS. For example, 15-Hz tACS suppressed MEPs (Zaghi et al., 2010), and 1.5 mA or 2 mA 20-Hz tACS reportedly increased MEP amplitudes (Gallasch et al., 2018; Wischnewski et al., 2019a). Therefore, after-effects induced by α- and β-tACS are inconsistent and weak. This is especially true for tACS delivered with small 1-mA currents, as opposed to larger currents, as suggested by a meta-analysis (Wischnewski et al., 2019b). Taken together, the suppressive effects of tACS on iTBS do not result from tACS after-effects but rather are generated through the interaction between iTBS and tACS.

### Limitations

There are several limitations to this study. First, neuronavigation was not available, and the TMS coil position was not monitored or recorded with a camera. However, the M1 hot spot was determined based on the standard protocol, and the position was marked with a pen to ensure the consistency of the TMS coil position throughout each session. Thus, we believe the TMS coil position variability to have been negligible. Second, the sample size was relatively small and slightly different between the α- and β-tACS experiments. However, this sample size was within the average range of other studies. Moreover, when the sample size in the α-tACS condition was limited to the first 13 subjects as in the β-tACS experiment, consistent results were obtained, i.e., a significant main effect of tACS condition (*F*_(2,257)_ = 4.66, *p* = 0.01). The differences were also significant between the peak and sham conditions (*F*_(1,167)_ = 4.4, *p* = 0.037) and between the peak and trough conditions (*F*_(1,167)_ = 9.58, *p* = 0.002), but not between the trough and sham conditions (*F*_(1,167)_ = 0.93, *p* = 0.34). Thus, it is unlikely that the difference in the sample size between the α- and β-tACS experiments caused the differential results. Third, iTBS effects are facilitatory in general, and this was observed for the β-tACS but not the α-tACS experiment. Because iTBS effects are known to vary among subjects (Hamada et al., 2013), individual variation could have influenced the data. Marginal effects can be found for other NIBS protocols such as cTBS (Goldsworthy et al., 2016). However, because the tACS effects were statistically demonstrated for combined stimulation compared with iTBS only (sham condition), it is likely that tACS suppressed the effects of iTBS. Finally, the present results were not achieved by the untransformed data, but after the data transformation, where MEP amplitudes were log-transformed to normalize their distribution and subtracted by baseline values to estimate the after-effects. Although these transformations were employed in other researchers (Avenanti et al., 2006; Goldsworthy et al., 2016; Borgomaneri et al., 2020), the tACS effect on iTBS was thought to be weak considering the data transformation as well as small η_*p*_^2^. Therefore, our results should be interpreted with caution.

## Conclusion

We explored the after-effects of iTBS synchronized with α- and β-tACS. We found phase-dependent suppression of iTBS at the peak phase for α-tACS, and phase-independent inhibition for β-tACS. We propose that cortical oscillations at α or β frequencies could interfere with iTBS activity through different mechanisms. Determining these differential mechanisms could provide new insights for understanding the mechanisms underlying iTBS, as well as other types of NIBS, and lead to more efficient protocols for enhancing cortical functions.

## Data Availability Statement

The raw data supporting the conclusions of this article will be made available by the authors, without undue reservation.

## Ethics Statement

The studies involving human participants were reviewed and approved by the Ethics Committee of Kyushu University and the International University of Health and Welfare. The patients/participants provided their written informed consent to participate in this study.

## Author Contributions

KO, HN, and ST developed the concept for the experiments. KO, HN, TI, and S-IO collected and analyzed the data. KO and HN drafted the manuscript. TI, S-IO, YG, and ST provided comments to improve the manuscript. All authors contributed to the article and approved the submitted version.

## Conflict of Interest

The authors declare that the research was conducted in the absence of any commercial or financial relationships that could be construed as a potential conflict of interest.

## Publisher’s Note

All claims expressed in this article are solely those of the authors and do not necessarily represent those of their affiliated organizations, or those of the publisher, the editors and the reviewers. Any product that may be evaluated in this article, or claim that may be made by its manufacturer, is not guaranteed or endorsed by the publisher.
